# Conventional vs. tunnelized facial artery myomucosal flaps in oral cavity reconstruction: a comparative analysis of outcomes and quality of life

**DOI:** 10.1007/s00405-026-10214-y

**Published:** 2026-04-23

**Authors:** Kasper Basse Reinholdt, Søren Dührr Gade, Arunas Pikelis, Tejs Ehlers Klug

**Affiliations:** 1 Department of Otorhinolaryngology, Head and Neck Surgery, Aarhus University Hospital, Palle Juul-Jensens Boulevard 99, Aarhus N, Aarhus, DK-8200 Denmark; 2 Aarhus University, Aarhus, Denmark

**Keywords:** Facial artery myomucosal flap, FAMM flap, Tunnelized FAMM flap, Buccal flap, Oral cancer, Oral cavity reconstruction

## Abstract

**Purpose:**

The facial artery myomucosal (FAMM) flap (conventional FAMM flap) and the tunnelized FAMM island flap (tunnelized FAMM flap) are established techniques for oral cavity reconstruction. However, evidence on complications rates is limited to small cohorts, and long-term quality of life (QoL) data are lacking. This study aimed to (1) determine the prevalence of complications, (2) identify risk factors for complications, and (3) compare QoL outcomes between patients with conventional and tunnelized flaps.

**Methods:**

This single-center, retrospective study included all patients, who underwent oral cavity cancer ablation with reconstruction using either a unilateral conventional or tunnelized FAMM flap between 2018 and 2025. Demographics, surgical details, complications, and EORTC QLQ-H&N35 scores at 12-, 24-, and 36-months were analyzed. Uni- and multivariate analyses were performed.

**Results:**

In total, 141 patients were included, reconstructed with either conventional (*n* = 114, 81%) or tunnelized (*n* = 27, 19%) FAMM flaps. Complications occurred in 18 patients (13%), including partial (*n* = 7) or total (*n* = 5) flap necrosis, hematoma (*n* = 5), and infection (*n* = 1). Tunnelized FAMM flaps were associated with significantly higher complication rates (37%) compared with those receiving a conventional FAMM flap (7%; *p* < 0.001). Flap type was the only statistically significant risk factor for complications in multivariate analyses. Long-term QoL outcomes were similar between groups.

**Conclusion:**

Based on our cohort and surgical techniques (without including the facial vein in the tunnelized FAMM flap), the conventional FAMM flap was a safe and reliable flap, while the tunnelized FAMM flap was associated with increased risk of complications and without QoL benefits.

## Introduction

The facial artery myomucosal (FAMM) flap, first described by Pribaz et al. in 1992 [1], is a well-established option for reconstruction of small to moderate oral cavity defects. Its advantages include tissue replacement of similar types, versatility, robustness, and low complication rates, donor site morbidity, and technical demands. Limitations of the original FAMM flap, such as the need for tooth extraction or second stage pedicle division, have prompted several modifications. One such technique was introduced by Duranceau et al. in 2011 [2], in which the anterior flap incision is prolonged to reach the oral cavity defect to insert the flap directly over the alveolar ridge, thus avoiding the need for a second stage procedure (referred to as “conventional” FAMM flaps). Another established modification, the tunnelized FAMM island flap (“tunnelized” FAMM flap), was first described for tongue reconstruction by Zhao et al. in 2003 [3] and developed and popularized by Massarelli et al. in 2008 [4]. This approach involves dissecting the facial vessels and tunnelling the islanded flap to the neck around the mandible and back into the oral cavity through the floor of the mouth (FOM), thereby adding the advantages of avoiding tooth extraction and prolonging the reach of the flap. Despite their theoretical advantages, existing evidence is largely limited to small cohorts and comparative data on complication rates and long-term QoL outcomes are lacking.

At our tertiary institution, conventional FAMM flaps have been used for decades and are routinely employed for reconstruction of FOM, alveolar ridge, and tongue defects. Despite their utility, the need for tooth extraction and limitations in reach persist. In recent years, we have therefore included the tunnelized FAMM flap as an alternative for selected cases. With the increasing number of head and neck cancer survivors [5], there is a growing focus on post-treatment morbidity and QoL. We hypothesized that tunnelized FAMM flaps, compared to conventional FAMM flaps, may offer advantages in QoL, particularly regarding dentition preservation and chewing function, while potentially carrying higher complication risks due to its technical demands and vascular fragility.

We aimed to (1) determine the prevalence of complications, (2) identify risk factors for complications, and (3) compare patients’ long-term QoL after oral cavity cancer resection with conventional vs. tunnelized FAMM flap reconstruction.

## Methods

### Study population

This retrospective cohort study included all patients who underwent oral cavity cancer surgery and reconstruction with either a conventional or tunnelized FAMM flap (also referred to as the conventional and tunnelized group) at the Department of Otorhinolaryngology, Head and Neck Surgery, Aarhus University Hospital between January 2018 and August 2025. Patients with bilateral FAMM flaps were not included (*n* = 6). Patients were selected for conventional FAMM flap reconstruction routinely by clinical indication, and tunnelized FAMM flap reconstruction were only considered for selected patients to avoid unnecessary extraction of healthy teeth. Data acquisition was managed using the Research Electronic Data Capture (REDCap) system. The study was approved by the Danish Data Protection Agency (#1-16-02-795-17). No ethical approval is required according to The Central Denmark Region Committees on Health Research Ethics.

### Data collection

The following variables were extracted from a prospectively maintained departmental database: patient age, gender, tobacco smoking status, alcohol consumption, Charlson comorbidity index (CCI), WHO performance status (PS), preoperative blood tests, tumour stage and location, history of head and neck radiotherapy, adjuvant radiotherapy, performed neck dissection, duration of surgery, flap type, secondary procedures, and postoperative complications. Patient-reported QoL measures were collected 12, 24, and 36 months postoperatively using the Danish version of the European Organization for Research and Treatment of Cancer Quality of Life Questionnaire Head and Neck 35 (EORTC QLQ-H&N35) [6].

### Surgical techniques

**Fig. 1:**
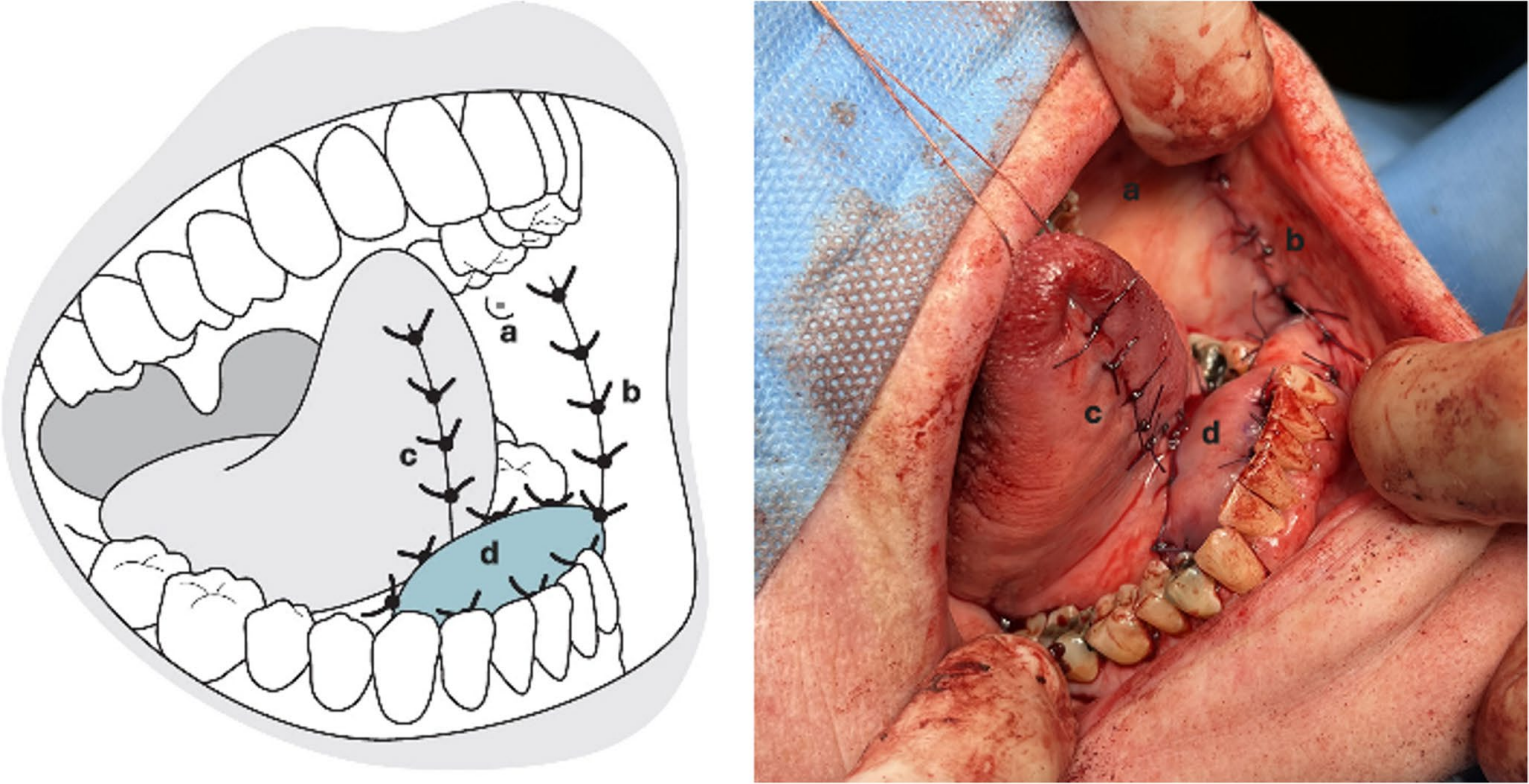
Conventional facial artery myomucosal (FAMM) flap reconstruction. a Parotid papillae; b Donor site; c Tongue; d FAMM flap

**Fig. 2:**
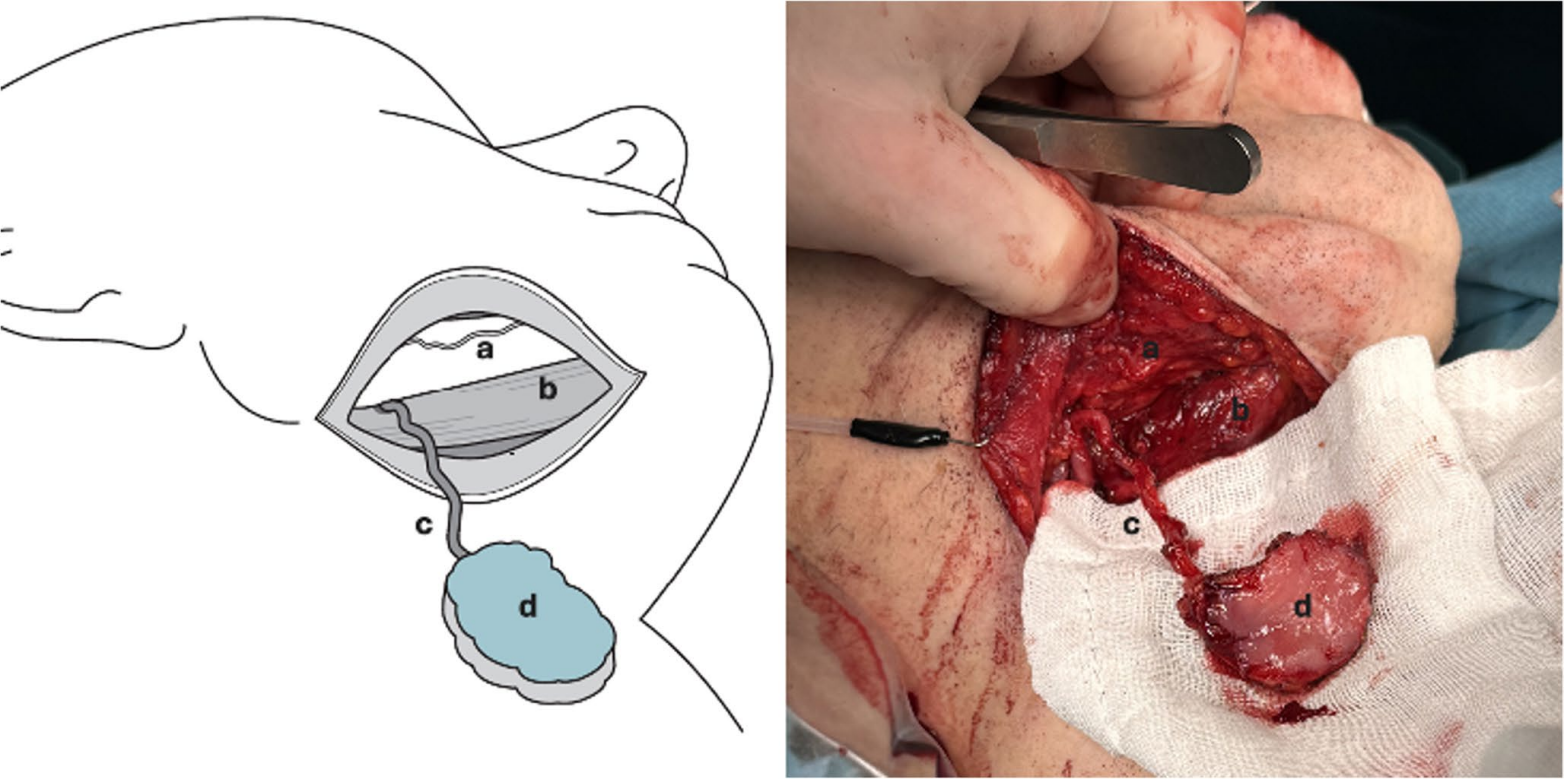
Tunnelized facial artery myomucosal (FAMM) flap reconstruction. a Marginal mandibular branch of facial nerve (CN VII); b Posterior belly of digastric muscle; c Facial artery; d FAMM flap

The conventional FAMM flaps were designed according to Duranceau et al. [2] (Fig. 1) and for tunnelized FAMM flaps as described by Massarelli et al. [4] (Fig. 2), with the exception that the facial vein was not included in the flap as originally described. Consequently, venous drainage relied on concomitant venous branches accompanying the facial artery. Donor sites were closed with primary suture or with buccal fat pad advancement. All patients were advised to pause antithrombotic therapy prior to treatment and all patients received perioperative prophylactic treatment with intravenous cefuroxime and metronidazole, typically continued for 5–7 days postoperatively, after which the patient was discharged. A nasogastric feeding tube was routinely placed and removed once sufficient oral healing allowed for initiation of oral intake. Suction drains were used in neck dissections but not intraorally. All patients were evaluated by an occupational therapist after surgery and instructed in rehabilitation exercises.

### Postoperative complications

Only clinically significant postoperative complications were included in the analysis. These were defined as events requiring medical or surgical intervention, such as reoperation, administration of antibiotics, or surgical revision (e.g., trimming of the flap in local anesthesia), corresponding to Clavien-Dindo (2004) grade II or higher.

### Statistical analysis

Continuous variables (age, smoking load, preoperative blood tests, duration of surgery, and QoL measures) were analyzed using Student’s *t*-test for independent samples, assuming normal distribution. Categorical variables (gender, history of radiotherapy, adjuvant radiotherapy, smoking status, alcohol use, tumour stage, neck dissection, flap type, complications, and QoL measures) were compared using Fisher’s exact test. Univariate regression analyses were performed to compare baseline characteristics between groups and flap complications. Uni- and multivariate analyses were made to identify risk factors for complications. A p-value of < 0.05 was considered statistically significant. All statistical analyses were performed using STATA software, version 15.1 (StataCorp LLC, College Station, TX, USA).

## Results

In total, 141 patients with either a unilateral conventional (*n* = 114, 81%) or tunnelized FAMM flap reconstruction (*n* = 27, 19%) were included (Table 1). All flaps were based on anterograde facial artery flow, except one conventional FAMM flap, which was constructed with retrograde flow. Patients mean age (60 vs. 67 years), smoking load (34 vs. 45 pack years), Charlson comorbidity index (41% vs. 19% CCI 0–3) and WHO performance status (59% vs. 26% PS 0) was lower, and the proportion of patients with tumour located on tongue was higher (37% vs. 12%) in the tunnelized group compared to the conventional group (Table 1). The other baseline characteristics were similar between groups.

**Table 1:** Clinical characteristics of patients with conventional or tunnelized facial artery myomucosal (FAMM) flap reconstruction

Variable	FAMM flap		
	Conventional (*n* = 114)	Tunnelized (*n* = 27)	*p*
*Age (years)*,* mean* (SD)	67.0 (9.4)	60.3 (11.2)	0.002^a^
*Gender*,* n (%)*			0.82^b^
Male	77 (68%)	19 (70%)	
Female	37 (32%)	8 (30%)	
*History of head and neck radiotherapy*,* n (%)*	17 (15%)	3 (11%)	0.77^b^
*Smoking status*,* n (%)*			
Current	79 (69%)	16 (59%)	0.36^b^
Previously	27 (24%)	7 (26%)	0.81^b^
Never	7 (6%)	3 (11%)	0.40^b^
Unknown	1 (1%)	1 (4%)	0.34^b^
*Smoking load*^*c*^ *(pack years)*,* mean (SD)*	45 (23)	34 (17)	0.041^a^
*Alcohol abuse*^*d*^ *(current/previous)*,* n (%)*	64 (56%)	13 (48%)	0.52^b^
*Charlson Comorbidity Index*			
0–3 (moderate risk)	22 (19%)	11 (41%)	0.024^b^
4–5 (high risk)	56 (49%)	11 (41%)	0.52^b^
≥ 6 (very high risk)	36 (32%)	5 (19%)	0.24^b^
*WHO Performance Status*,* n (%)*			
0	30 (26%)	16 (59%)	0.002^b^
1	68 (60%)	10 (37%)	0.051^b^
2–4	16 (14%)	1 (4%)	0.20^b^
*Pre-operative blood tests*,* mean (SD)*			
Serum albumin (g/L)	39.1 (3.5)	40.4 (4.3)	0.10^a^
Haemoglobin (mmol/L)	8.6 (1.5)	8.4 (1.9)	0.52^a^
*Tumour location*,* n (%)*			
Floor of mouth	79 (69%)	17 (63%)	0.65^b^
Tongue	14 (12%)	10 (37%)	0.004^b^
Alveolar ridge	16 (14%)	-	0.042^b^
Retromolar trigone	5 (4%)	-	0.58^b^
*pT-stage (UICC 8)*,* n (%)*			
T1	60 (53%)	12 (44%)	0.52^b^
T2	31 (27%)	7 (26%)	1.00^b^
T3	19 (17%)	7 (26%)	0.28^b^
T4a	4 (4%)	1 (4%)	1.00^b^
*Neck dissection including level I*,* n (%)*	107 (94%)	27 (100%)	0.31^b^
*Duration of surgery (min)*,* mean (SD)*	359 (87)	355 (57)	0.84^a^
*Adjuvant radiotherapy*,* n (%)*	21 (18%)	8 (30%)	0.20^b^

^a^Students *t*-test

^b^Fisher’s exact test

^c^One pack year: Smoking 20 cigarettes per day for one year

^d^Current or former significant alcohol intake for minimum one year (defined as women: >14 units per week; men: >21 units per week). One unit is 12 g of alcohol. Unknown status for 17 patients with FAMM flaps

Abbreviations: *SD,* standard deviation

Tooth extraction was performed in 72% (82/114) cases using conventional FAMM flap vs. 4% (1/27) of tunnelized FAMM flap cases (*p* < 0.001). Ipsilateral neck dissection including level I was performed in 134 (95%) cases, most commonly (126/134, 94%) standard ipsilateral level I-III neck dissection, while sentinel node biopsy was performed in five (4%) cases, all of which also included dissection of ipsilateral level I. No neck surgery was performed in seven (5%) cases. Secondary adjusting surgery with pedicle division/vestibuloplasty or release of buccal or tongue fibrosis was performed in 7% (8/114) following conventional and 15% (4/27) of patients following tunnelized FAMM flap reconstruction (*p* = 0.24).

### Postoperative complications and risk factors

Complications were observed in 13% (18/141) of cases (Table 2). Tunnelized FAMM flaps were significantly associated with higher complication rates (37%, 10/27) compared with conventional FAMM flaps (7%, 8/114; *p* < 0.001). The rate of total or partial flap necrosis was significantly higher in the tunnelized group (11% and 19%, respectively) compared to the conventional group (2% and 2%; *p* = 0.048 and *p* = 0.003, respectively). All defects secondary to flap necrosis (total: *n* = 5, or partial: *n* = 7) healed uneventfully by secondary granulation and without exposure of bone. Hence, no patients underwent surgery because of flap necrosis, except for removal of necrotic tissue. In the subgroup with FAMM flap reconstruction and level I neck dissection, persistent marginal mandibular nerve palsy at 12 months was documented in 4/107 (4%) patients in the conventional group and 3/27 (11%) in the tunnelized group (*p* = 0.15).

**Table 2:** Postoperative complications in patients with conventional or tunnelized facial artery myomucosal (FAMM) flap reconstruction

		FAMM flap			
Variable, n (%)	Total (*n* = 141)	Conventional (*n* = 114)	Tunnelized (*n* = 27)	OR (95% CI)	*p* ^a^
Total flap necrosis	5 (4%)	2 (2%)	3 (11%)	7.0 (0.74-86.3)	0.048
Partial flap necrosis	7 (5%)	2 (2%)	5 (19%)	12.7 (1.9-138)	0.003
Haemorrhage^b^ (oral)	5 (4%)	3 (3%)	2 (7%)	3.0 (0.23-27.0)	0.24
Infection (oral)	1 (1%)	1 (1%)	0	-	1.00
No complications	123 (87%)	106 (93%)	17 (63%)	0.13 (0.04-0.43)	<0.001
Total complications	18 (13%)	8 (7%)	10 (37%)	7.8 (2.3-25.8)	<0.001

^a^Fisher’s exact test

^b^Requiring surgery

Abbreviations: *OR,* odds ratio; *CI,* confidence interval

In univariate analysis, the following parameters were associated with complications: lower age (mean 61 vs. 66 years, *p* = 0.027), low CCI (0–3) (20% vs. 44%, *p* = 0.036), and low PS (0) (29% vs. 56%, *p* = 0.033), FAMM flap type (tunnelized, *p* < 0.001) (Table 3). In multivariate analysis, flap type was the only statistically significant parameter associated with complications (*p* = 0.009, when adjusting for age, CCI, and PS). This association maintained statistical significance also adjusting for adjuvant radiotherapy (*p* = 0.011), and tumour site (*p* = 0.015).

**Table 3:** Clinical characteristics of 141 patients undergoing oral cavity cancer resection with conventional or tunnelized facial artery myomucosal (FAMM) flap reconstruction stratified by the presence or absence of complications. Odds ratios (OR) for complications are calculated for each variable

Variable	Complications (*n* = 18)	No complications (*n* = 123)	OR (95% CI)	*p*
*Age (years), mean* (SD)	60.8 (11.2)	66.4 (9.8)		0.027^a^
*Gender, n (%)*			1.25 (0.38-4.8)	0.79^b^
Male	13 (72%)	83 (67%)		
Female	5 (28%)	40 (33%)		
*History of head and neck radiotherapy, n (%)*	2 (11%)	18 (15%)	0.73 (0.08-3.6)	1.00^b^
*Smoking status, n (%)*				
Current	13 (72%)	83 (67%)	1.25 (0.38-4.8)	0.79^b^
Previously	3 (17%)	31 (25%)	0.59 (0.10-2.3)	0.56^b^
Never	2 (11%)	8 (7%)	1.80 (0.17-10.1)	0.62^b^
Unknown	-	1 (1%)	-	1.00^b^
*Smoking load* ^*c*^ * (pack years), mean (SD)*	46 (35)	42 (20)		0.54^a^
*Alcohol abuse* ^*d*^ * (current/previous), n (%)*	10 (56%)	67 (54%)	1.04 (0.34-3.3)	1.00^b^
*Charlson Comorbidity Index*				
0-3 (moderate risk)	8 (44%)	25 (20%)	3.1 (0.96-9.8)	0.036^b^
4-5 (high risk)	8 (44%)	57 (46%)	0.93 (0.30-2.8)	1.00^b^
≥ 6 (very high risk)	2 (11%)	41 (33%)	0.25 (0.03-1.2)	0.06^b^
*WHO Performance Status, n (%)*				
0	10 (56%)	36 (29%)	3.0 (0.98-9.5)	0.033^b^
1	7 (39%)	71 (58%)	0.47 (0.14-1.4)	0.20^b^
2-4	1 (6%)	16 (13%)	0.39 (0.01-2.9)	0.70^b^
*Pre-operative blood tests, mean (SD)*				
Serum albumin (g/L)	40.7 (3.3)	39.2 (3.8)		0.11^a^
Haemoglobin (mmol/L)	8.9 (1.0)	8.6 (1.7)		0.36^a^
*FAMM-flap type, n (%)*			7.8 (2.3-25.8)	<0.001^b^
Conventional	8 (44%)	106 (86%)		
Tunnelized	10 (56%)	17 (14%)		
*Tumour location, n (%)*				
Floor of mouth	14 (78%)	82 (67%)	1.62 (0.47-7.2)	0.58^b^
Tongue	4 (22%)	20 (16%)	1.47 (0.32-5.4)	0.51^b^
Alveolar ridge	-	16 (13%)	-	0.22^b^
Retromolar trigone	-	5 (4%)	-	1.00^b^
*pT-stage (UICC 8), n (%)*				
T1	7 (39%)	65 (53%)	0.57 (0.18-1.7)	0.32^b^
T2	6 (33%)	33 (27%)	1.36 (0.39-4.3)	0.58^b^
T3	5 (28%)	20 (16%)	1.98 (0.49-6.8)	0.32^b^
T4a	-	5 (4%)	-	1.00^b^
*Neck dissection including level I, n (%)*	17 (94%)	117 (95%)	0.87 (0.10-42.4)	1.00^b^
*Duration of surgery (min), mean (SD)*	348 (69)	359 (84)		0.59^a^

^a^Student´s t-test

^b^Fisher’s exact test

^c^One pack year: Smoking 20 cigarettes per day for one year

^d^Current or former significant alcohol intake for minimum one year (defined as women: >14 units per week; men: >21 units per week). One unit is 12 grams of alcohol. Unknown status for 17 patients with FAMM flaps

Abbreviations: *OR,* odds ratio; *SD,* standard deviation; *CI,* confidence interval

### Quality of life outcomes

Disease-specific QoL scores (EORTC QLQ-H&N35) 12, 24, and 36 months are summarized in Table 4. Response rates were 63% (59/94) and 60% (15/25) at 12 months follow-up, 51% (40/79) and 73% (16/22) at 24 months follow-up, and 47% (28/60) and 68% (13/19) at 36 months follow-up in the conventional and tunnelized group, respectively.

**Table 4:** Oral cavity cancer patients´ quality of life measured as EORTC QLQ-H&N35^a^ scores 12-^b^, 24-^c^, and 36-months^d^ following surgery with conventional or tunnelized facial artery myomucosal (FAMM) flap reconstruction

		FAMM flap		
Parameter	All	Conventional	Tunnelized	*p* ^e^
*Pain, mean (SD)*				
12 months	19.0 (28.7)	19.0 (27.3)	20.0 (34.3)	0.77
24 months	16.0 (25.0)	16.3 (26.0)	15.0 (23.0)	0.72
36 months	16.0 (23.0)	18.0 (25.0)	12.3 (23.0)	0.17
*Swallowing, mean (SD)*				
12 months	19.3 (29.7)	19.7 (29.3)	18.3 (31.7)	0.75
24 months	19.7 (30.3)	22.3 (32.7)	13.0 (22.0)	0.039
36 months	18.3 (28.7)	18.0 (28.0)	19.0 (30.7)	0.87
*Teeth, mean (SD)*				
12 months	30.3 (39.0)	34.3 (40.0)	13.3 (30.3)	0.061
24 months	25.3 (36.7)	29.0 (37.3)	16.7 (34.3)	0.26
36 months	22.7 (30.7)	27.0 (32.0)	12.7 (25.7)	0.17
*Opening mouth, mean (SD)*				
12 months	26.7 (34.3)	27.7 (34.7)	22.3 (35.0)	0.59
24 months	23.7 (30.3)	26.7 (33.7)	16.7 (17.3)	0.27
36 months	23.7 (32.0)	25.0 (32.3)	20.7 (32.0)	0.68
*Dry mouth, mean (SD)*				
12 months	44.7 (39.0)	45.7 (39.0)	40.0 (51.0)	0.61
24 months	39.3 (34.7)	41.0 (35.3)	35.3 (33.3)	0.59
36 months	34.3 (33.3)	33.3 (32.7)	36.0 (36.0)	0.81
*Sticky saliva, mean (SD)*				
12 months	31.7 (36.3)	32.7 (38.7)	26.7 (25.7)	0.57
24 months	35.7 (35.3)	36.0 (38.7)	35.7 (26.7)	0.98
36 months	23.3 (30.3)	24.7 (32.7)	20.0 (23.3)	0.68
*Senses, mean (SD)*				
12 months	24.0 (31.7)	25.7 (33.0)	16.7 (24.3)	0.16
24 months	17.0 (28.3)	15.0 (26.3)	22.0 (32.3)	0.25
36 months	18.7 (24.0)	19.0 (24.7)	17.3 (23.7)	0.77
*Coughing, mean (SD)*				
12 months	21.3 (29.3)	22.0 (31.3)	17.7 (21.3)	0.62
24 months	11.0 (19.3)	11.0 (20.7)	10.3 (16.0)	0.90
36 months	12.7 (19.7)	13.0 (21.0)	11.0 (16.3)	0.77
*Felt ill, mean (SD)*				
12 months	12.0 (23.0)	12.0 (22.0)	13.3 (27.7)	0.83
24 months	6.7 (15.0)	6.0 (15.0)	8.3 (15.0)	0.60
36 months	9.0 (22.3)	12.0 (26.0)	2.7 (9.3)	0.22
*Speech, mean (SD)*				
12 months	21.0 (27.3)	22.0 (28.7)	17.0 (21.0)	0.27
24 months	20.0 (27.0)	19.7 (27.7)	21.7 (26.3)	0.68
36 months	16.3 (23.7)	16.0 (25.0)	17.0 (20.3)	0.82
*Social eating, mean (SD)*				
12 months	36.0 (38.0)	38.0 (39.0)	27.3 (33.3)	0.047
24 months	30.7 (37.3)	34.3 (38.3)	22.3 (33.0)	0.033
36 months	28.3 (34.3)	28.0 (34.7)	29.0 (34.3)	0.88
*Social contact, mean (SD)*				
12 months	16.7 (30.3)	17.3 (31.7)	14.3 (24.7)	0.45
24 months	14.7 (27.7)	13.7 (28.0)	17.7 (26.7)	0.30
36 months	13.3 (25.7)	10.7 (22.7)	19.7 (30.7)	0.020
*Sexuality, mean (SD)*				
12 months	13.3 (34.0)	10.3 (24.7)	23.3 (34.0)	0.023
24 months	8.3 (19.7)	7.0 (17.7)	11.3 (24.0)	0.31
36 months	22.3 (35.0)	21.0 (33.3)	25.0 (38.3)	0.65
*Pain killers, yes, n (%)*				
12 months	36/73 (49%)	27/58 (47%)	9/15 (60%)	0.40
24 months	27/56 (48%)	20/40 (50%)	7/16 (44%)	0.77
36 months	16/41 (39%)	12/28 (43%)	4/13 (31%)	0.51
*Nutritional supplements, yes, n (%)*				
12 months	29/74 (39%)	26/59 (44%)	3/15 (20%)	0.14
24 months	16/54 (30%)	11/38 (29%)	5/16 (31%)	1.00
36 months	15/39 (38%)	10/26 (38%)	5/13 (38%)	1.00
*Feeding tube, yes, n (%)*				
12 months	3/72 (4%)	2/58 (3%)	1/14 (7%)	1.00
24 months	6/55 (11%)	5/38 (13%)	1/16 (6%)	0.66
36 months	2/37 (5%)	0/26 (0)	2/11 (18%)	0.08
*Weight loss, yes, n (%)*				
12 months	9/62 (15%)	7/47 (15%)	2/15 (13%)	1.00
24 months	6/48 (13%)	5/33 (15%)	1/15 (7%)	0.65
36 months	4/35 (11%)	3/24 (13%)	1/11 (9%)	1.00
*Weight gain, yes, n (%)*				
12 months	13/62 (21%)	11/47 (23%)	2/15 (13%)	0.49
24 months	9/48 (19%)	6/33 (18%)	3/15 (20%)	1.00
36 months	6/35 (17%)	3/24 (13%)	3/11 (27%)	0.35

^a^According to EORTC QLQ-H&N35 version 1 (www.eortc.org). Scores range 0–100, with higher scores indicating greater symptom burden

^b^12 month follow-up data on eligible patients (i.e., operated more than one year prior to study end) were available for 59/94 patients (63%) in the conventional group and 15/25 (60%) in the tunnelized group

^c^24 month follow-up data on eligible patients (i.e., operated more than two years prior to study end) were available for 40/79 (51%) in the conventional group and 16/22 (73%) in the tunnelized group

^d^36 month follow-up data on eligible patients (i.e., operated more than three years prior to study end) were available for 28/60 (47%) in the conventional group and 13/19 (68%) in the tunnelized group

^e^Students’ t-test for continuous variables and Fischer’s exact test for categorical variables

Abbreviations: *SD*, standard deviation

No statistically significant differences were found in most parameters at all three follow-ups, except for the following: tunnelized FAMM flap patients reported significantly more sexual problems at 12 months (*p* = 0.023) and greater social contact difficulties at 36 months (*p* = 0.020). Furthermore, problems with swallowing at 24 months (*p* = 0.039) and social eating at 12 and 24 months (*p* = 0.047 and *p* = 0.033, respectively) were significantly higher in the conventional group compared to the tunnelized group. Overall health-status and overall QoL tended to be better in the tunnelized group compared to the conventional group at all follow-ups, though not reaching significant values (Table 5).

**Table 5:** Oral cavity cancer patient´s overall health status and quality of life 12-, 24-, and 36-months following surgery with conventional or tunnelized facial artery myomucosal (FAMM) flap reconstruction

		FAMM flap		
Parameter, n (%)	All	Conventional	Tunnelized	*p* ^a^
*Overall health status*				
12 months				1.00
Good or very good	35/71 (49%)	26/57 (46%)	9/14 (64%)	0.25
Bad or very bad	7/71 (10%)	5/57 (9%)	2/14 (14%)	0.62
Unknown	29/71 (41%)	26/57 (46%)	3/14 (21%)	0.13
24 months				0.53
Good or very good	31/54 (57%)	20/39 (51%)	11/15 (73%)	0.22
Bad or very bad	3/54 (6%)	3/39 (8%)	0/15 (0%)	0.55
Unknown	20/54 (37%)	16/39 (41%)	4/15 (27%)	0.37
36 months				0.53
Good or very good	27/40 (68%)	18/28 (64%)	9/12 (75%)	0.72
Bad or very bad	3/40 (8%)	3/28 (11%)	0/12 (-)	0.54
Unknown	10/40 (25%)	7/28 (25%)	3/12 (25%)	1.00
*Overall quality of life*				
12 months				1.00
Good or very good	32/70 (46%)	25/56 (45%)	7/14 (50%)	0.77
Bad or very bad	9/70 (13%)	7/56 (13%)	2/14 (14%)	1.00
Unknown	29/70 (41%)	24/56 (43%)	5/14 (36%)	0.77
24 months				0.08
Good or very good	32/54 (59%)	21/39 (54%)	11/15 (73%)	0.23
Bad or very bad	8/54 (15%)	8/39 (21%)	0/15 (-)	0.09
Unknown	14/54 (26%)	10/39 (26%)	4/15 (27%)	1.00
36 months				0.13
Good or very good	18/33 (55%)	11/24 (46%)	7/9 (78%)	0.13
Bad or very bad	6/33 (18%)	6/24 (25%)	0/9 (-)	0.16
Unknown	9/33 (27%)	7/24 (29%)	2/9 (22%)	1.00

^a^Fischer’s exact test

Baseline characteristics (including flap type) were comparable between responders and non-responders, except for previous head and neck radiotherapy, which was more common for non-responders than responders (24% vs. 8%, *p* = 0.028).

Four sub-analyses were performed on 12-month QoL data: (1) Focusing on dental problems and eating difficulties, patients in the conventional group, who had tooth extraction during surgery (*n* = 82) were compared with patients in the tunnelized group without tooth extraction (*n* = 26). Patients with tooth extraction had significantly lower QoL in questions related to teeth (*p* = 0.025) and tended to have lower QoL related to eating (*p* = 0.28) compared to patients without tooth extraction. (2) Patients with partial or total flap necrosis were compared with patients with no flap necrosis, and all parameters were comparable, except for higher pain scores and sexuality problems in patients with flap necrosis (*p* = 0.037 and *p* = 0.047, respectively). Sub-analyses for the 24- and 36-month follow-up data were not conducted due to limited data.

## Discussion

The overall complication rate among 141 FAMM flap oral reconstructions was 13%, with tunnelized FAMM flaps demonstrating significantly higher complication rates compared to conventional FAMM flaps (37% vs. 7%). Total flap necrosis occurred more frequently in tunnelized FAMM flaps (11% vs. 2%). Notably, no cases of flap failure required secondary reconstructive procedures, as healing by secondary intention occurred in all cases. Nevertheless, flap loss may still result in functional sequelae, including progressive contracture and restricted tongue mobility. Importantly, flap necrosis did not appear to adversely affect overall QoL, with exception of higher pain scores at 12-month follow-up.

Our findings are consistent with a recent systematic review by Mattey et al., which analyzed 407 FAMM flap reconstructions and reported an overall flap failure rate of 1.7% [7]. The review documented a 26% non-failure complication rate, most commonly partial necrosis (6%), wound dehiscence (4%), and venous congestion (3%). However, the authors did not distinguish between FAMM flap types, and no analyses of risk factors were conducted. An earlier systematic review by Ayad et al. stratified complication rates according to flap types and concluded that FAMM flaps are generally safe and versatile, although heterogeneity in reported outcomes hindered comparison of complication rates [8]. The authors further highlighted the need for larger studies to adequately evaluate complication profiles across flap designs. To our knowledge, the present study is the largest to date to substantiate the high safety profile of conventional FAMM flaps and to raise important considerations regarding the routine use of tunnelized FAMM flaps without including the facial vein.

Compromised venous drainage from the flap appears to be the main contributing factor to flap necrosis, and techniques preserving the facial vein, as originally described by Zhao [3] and Massarelli [4], may mitigate this risk. Massarelli et al. reported no major complications in 27 tunnelized FAMM flaps when the facial vein was included [9], which was later supported by animal studies [10]. However, preserving the facial vein demands high surgical expertise, and if the facial vein cannot be preserved, a conventional FAMM flap or alternative reconstructions may be considered.

The complication rates associated with conventional FAMM flaps compare favorably with those reported for alternative reconstructive options for medium sized oral defects, including the submental island flap [11], radial forearm (RFFF) and anterolateral thigh (ALT) free flaps [12]. Moreover, when compared to RFFF, Ibrahim et al. found that FAMM flaps were associated with lower costs, shorter operating time, and similar speech and swallowing outcomes [13].

### Risk factors for postoperative complications

FAMM flap type (tunnelized) was the only variable that was significantly associated with complications in multivariate analysis. Although younger age, lower CCI, and lower PS were significantly associated with higher complication rates in univariate analyses, these parameters lost their statistical significance, when adjusting for flap type. In addition, the direction of these associations was clinically counterintuitive, and we interpret these variables as confounders by indication. To date, no previous studies have identified risk factors that guide the selection of FAMM flap types. It has been suggested that prior head and neck radiotherapy or neck dissection may contraindicate FAMM flap reconstruction. However, our findings suggest that neither represents an absolute contraindication. Nevertheless, while previous studies have shown that preservation of facial vessels does not compromise oncologic safety in cN0 patients [14, 15], even in cases subsequently upstaged to pN+, particular care should be taken when considering FAMM flap reconstruction in patients with cN+ disease. This is especially true when metastatic involvement of level 1B/perifacial lymph nodes necessitate sacrifice of the facial vessels, which could otherwise jeopardize oncologic safety. In these cases, alternative reconstructive strategies, such as a contralateral tunnelized FAMM flap or free microvascular flap, should be planned as backups.

### Quality of life analysis

In the present study, postoperative QoL assessed using EORTC QLQ-H&N35 revealed moderate symptom burden across most domains. Compared with normative data from healthy individuals - typically scoring below 10 across domains - patients undergoing FAMM flap reconstruction demonstrated higher scores on all domains, aligning with previous reports of elevated symptom burden among head and neck cancer survivors in general, even years after treatment [16]. Both statistically and clinically significant advantages were noted for the tunnelized FAMM flap in *swallowing* at 24 months (mean 13.0 vs. 22.3) and *social eating* at both 12 and 24 months (mean 27.3 vs. 38.0 and 22.3 vs. 34.3, respectively). These differences, ranging from 9 to 12 points, surpass the threshold for moderate clinical relevance [17]. A large early difference was also noted for the *teeth* domain at 12 months (> 20 points), suggesting better dental comfort with the tunnelized FAMM flap, although this did not reach statistical significance (*p* = 0.061). However, when comparing patients with vs. without tooth extractions, the benefits of tooth-sparing approaches reached statistical significance (*p* < 0.025), but these analyses were post hoc, underpowered, and unadjusted for multiple testing and should be regarded as exploratory. Few studies have investigated QoL after FAMM flap reconstruction. Recent studies demonstrated satisfactory 12 months QoL outcomes following various FAMM flap techniques (including conventional and tunnelized), though these analyses did not stratify results by flap type and were limited by short follow-up durations [9, 18]. In this regard, the current study offers novel and long-term insights into QoL and associated differences between conventional and tunnelized FAMM flaps, suggesting that both approaches yield favorable and broadly comparable functional outcomes.

### Strength and limitations

The strengths of this study include the use of a large, consecutive cohort with long-term follow-up and a prospectively maintained clinical database, which together yielded robust outcome data supported by relatively high follow-up response rates. Additionally, complications and QoL data were comprehensively reported using validated instruments such as Clavien-Dindo grading and QLQ-H&N35, facilitating comparison with future research.

Several limitations, however, warrant consideration. Although routine quality checks were performed, the possibility of data entry errors cannot be entirely excluded. Similarly, operative time is difficult to compare between groups and across studies because it is influenced by concurrent procedures, including the extent of neck dissection, sentinel lymph node biopsy, and whether surgery was unilateral or bilateral. External validity is compromised by patient selection and the surgical technique used for tunnelized FAMM flaps in which the facial vein is not included as originally described. Confounding by indication must also be acknowledged: tunnelized FAMM flaps were primarily used in younger, healthier patients with good dentition. As a result, QoL comparison between flap types should be interpreted with caution. Differences in baseline QoL, as well as varying degrees of symptom awareness or sensitivity to physical and psychological changes, may contribute to differential reporting between groups. Moreover, the substantial overall symptom burden of head and neck cancer patients may obscure potential QoL advantages attributable to flap design. Since numerous QoL domains are tested across three time points some significant differences between groups may be random and of uncertain clinical relevance and should therefore be interpreted as exploratory. Even though no relevant differences were found between responders and non-responders, attrition and response bias must be acknowledged. Therefore, QoL comparisons should be regarded as hypothesis-generating and not interpreted as causal.

It is well established that key oral functions such as speech and swallowing are influenced by the subsite of resection, with central (oral tongue) and posterior (oropharyngeal) defects associated with the poorest outcomes [19]. In our cohort, tunnelized FAMM flaps were used more frequently for tongue reconstruction than conventional FAMM flaps. This distribution may therefore mask or attenuate potential differences in speech and swallowing outcomes between the two flap types.

## Conclusion

Our study suggests that the conventional FAMM flap is a safe and reliable option associated with low complication rates and satisfactory QoL in patients undergoing resection of small- to medium-sized oral cancers. In our cohort, the tunnelized FAMM flap without facial vein inclusion was associated with higher complication rates and modest, if any, QoL advantage, and therefore should be reserved for selected cases.

## Data Availability

Data is available from the corresponding author on request.
